# Life experiences of patients who have completed tuberculosis treatment: a qualitative investigation in southeast Brazil

**DOI:** 10.1186/1471-2458-13-595

**Published:** 2013-06-19

**Authors:** Ana Angélica Lima Dias, Daniela Maria Falcão de Oliveira, Egberto Ribeiro Turato, Rosely Moralez de Figueiredo

**Affiliations:** 1 Nursing Post-Graduation Program, Federal University of São Carlos (UFSCar), Rod. Washington Luís, Km 235, São Carlos, SP, Brazil; 2 Department of Medical Psychology and Psychiatry, Laboratory of Clinical-Qualitative Research, State University of Campinas (UNICAMP), Cidade Universitária, Campinas, SP, Brazil

**Keywords:** Tuberculosis, Cost of illness, Therapeutics, Qualitative research

## Abstract

**Background:**

Despite being curable, tuberculosis is still a stigmatized disease. Not only is TB patients’ suffering due to its clinical manifestations, but also because of society’s prejudice, embarrassing situations, and even self-discrimination. This study aims to investigate psychosocial experiences of patients who have completed tuberculosis treatment in São Carlos a municipality in the interior of São Paulo State, Brazil.

**Methods:**

This study, of a clinical-qualitative nature, sought to understand the meanings provided by the participants themselves. Fifteen individuals, who had successfully completed tuberculosis treatment, participated in this research. The sample size was established using the information saturation criterion. Data were collected by means of interviews with in-depth open-ended questions. Data were treated by categorizing and analyzing content according to themes.

**Results:**

Regardless of all progress, this study found that TB still causes patients to suffer from fear of transmission, social prejudice, and death. Despite the fact that the emotional support provided by families and healthcare professionals is considered essential to treatment adherence and completion, participants in this study reveal that friends and colleagues have distanced themselves from them for fear of contagion and/or prejudice. Ignorance about the disease and its transmission modes can be found in the interviewees’ statements, which seems to indicate that they have become vectors of transmission of stigma themselves. Patients’ medical leave from work during treatment may be due to both their health conditions and their attempt to avoid social/emotional embarrassment. There are accounts that TB has caused psychosocial damage to patients’ lives and that they feel more fatigue and lassitude and have begun to pay more attention to their own health.

**Conclusions:**

Healthcare workers should be aware of the ways TB treatment affect patients’ psychosocial life and develop strategies to mitigate these effects and provide opportunities for them to share their anxiety, suffering, and bio-psychosocial changes. In addition, healthcare professionals should seek to educate and, as a result, empower TB patients and their families with regard to this disease so as to break the existing vicious cycle of misinformation and prejudice.

## Background

Tuberculosis (TB) is an important public health problem worldwide [1]. WHO (World Health Organization) estimates that 2 billion people, or a third of the world’s population, are infected with *M. tuberculosis*. Roughly eight million of these individuals develop the disease and three million die from it every year [2].

Aimed at reducing morbidity and mortality from TB, DOTS (Directly Observed Treatment Short-Course) is highly recommended by WHO to control the disease. Moreover, DOTS is recommended because of its goal of detecting 70% of all new smear positive cases and its 85% success rate when implemented. In order to meet targets, this strategy comprises a set of actions, e.g., sputum smear microscopy among symptomatic patients, directly observed treatment (DOT), monitored and standardized, regular provision of drugs, information and registration system, and even the government’s commitment to prioritize TB among health policies [1].

Brazil has high TB incidence, which places it among the 22 countries most affected by this disease. In order to broaden and strengthen disease-control strategies, the country has established the *Programa Nacional de Controle da Tuberculose* (PNCT; National TB Control Program) anchored in decentralization and flattening of TB surveillance, prevention and control mechanisms. PNCT guarantees free distribution of drugs and supplies needed for TB prevention and control, providing citizens with universal access to health services for TB treatment [1].

Despite TB being a curable disease in almost all of new cases [1], professionals such as psychologists, anthropologists, sociologists, and TB analysts have repeatedly indicated that talking about the disease still causes discomfort and unease in the population, especially within the poorer communities [3].

Understanding the meanings conferred to TB by patients within their social settings enables one to approach this disease beyond clinical and conventional care. Patients’ lifestyles and thinking modes have an effect on how they respond to healthcare workers’ interventions and actions. However, this discussion is usually limited to evaluating these responses from the healthcare professionals’ standpoint, thereby disregarding how patients see their own condition [4].

TB is surrounded by intense grief, with implications to different spheres of life, including social relationships. This is due to long-standing negative representations about this disease, which result in stigma and discrimination [5]. Since Ancient Greece, the term ‘stigma’ has stood for bodily signs denoting something rare and evil about the signifier’s moral standing [6].

From patients’ and healthcare professionals’ perspective, TB dimensions indicate that besides its physical impact on patients’ lives, this disease has also a strong emotional impact, given that debilitating symptoms evoke disability, impotence, and self-discrimination [7].

In view of this emotional, social, and economic impasse, social/emotional support is crucial to treatment completion. This support can be accomplished by means of patients’ interaction with their families and friends, volunteers, and healthcare professionals and by promoting patients’ self-esteem, trust, and sense of belonging [8].

The authors of this study presuppose that eliciting TB patients experiences, from diagnosis to completion of treatment, is extremely relevant to better choosing actions that can improve their quality of life, increasing their adherence to treatment, minimizing undesirable results, facilitating the resumption of their daily lives, and promoting a shared language amongst health professionals and patients.

Therefore, this study aimed to investigate psychosocial experiences reported by patients who had successfully undergone TB treatment in São Carlos, São Paulo State – Brazil.

## Methods

This study – of a clinical-qualitative nature – adopted a humanistic design in that it sought to scientifically interpret the meanings participants conferred to their life experiences in their natural settings [9]. Qualitative researchers study phenomena in their natural settings, in an attempt to interpret it in terms of the meanings people confer to them [9,10]. Qualitative methods have their own specificities as regards sample composition, data analysis, and possible generalizations from results [10].

The research method utilized in this study is known as clinical-qualitative method. It is considered to be a particularization and refinement of generic qualitative methods used in the human sciences, herein applied to a healthcare context [9]. Data were collected by means of interview with open-ended questions. This approach – appropriate for qualitative research in the field of healthcare according to the literature [11] – was adopted in order to ensure in-depth interviews with the participants [9].

The sampling method utilized in qualitative research designs [9,10] involves the intentional search for individuals who possess information about the matter in question and are able to articulate it. Data is produced with the purpose of reformulating, deflecting, complementing and/or helping to clarify initial assumptions, as is desirable in any scientific construction.

The sample in this study consisted of patients that had successfully finished their TB treatment, in São Carlos, São Paulo State – Brazil. The following selection criteria were established: patients had undergone TB treatment for the first time; did not have HIV/AIDS; had completed treatment within the allotted time (six months); were older than 16 years; were intellectually, emotionally, and physically capable of undergoing a clinical-psychological research interview; and had agreed to participate in the study (signed a declaration of consent).

Transcripts from the interviews composed the study corpus, which was then subjected to qualitative content analysis according to themes [12]. Free-floating readings of the interviewees’ answers were conducted by the researchers so as to acquaint themselves with the material.

After applying the categorization strategy, the categories for this study were selected. The phenomena thus identified can then be interpreted to generate concepts that can be generalized to other settings [9,10]. The researchers made use of a theoretical framework based on the basic concepts of Health Psychology, such patients’ usual psychocultural adaptive handling of unfavorable events in their lived experiences.

The saturation criterion was employed to limit the size of the sample. In other words, it appeared clear to the researchers that after interviewing 15 patients, further interviews would contribute little with regard to the objectives initially set for this study [10].

This project was approved by the Committee of ethics in research with Human Beings of Federal University of São Carlos, process N° 413/2010.

The limitations of this study are inherent to qualitative methods. That is, this is a scientific study that advances concepts that can be generalized by its readers when they compare their lived situations to similar ones depicted in this research, i.e., they engage in naturalistic generalization or comprehension by resemblance.

## Results and Discussion

### Sample characterization

Participants had mainly pulmonary disease (80%), mean age of 47 years (ranging from 16 to 80 years), and were predominantly male (80%). Ten of them (67%) had eight or more years of schooling, thirteen (86%) lived with their families, and nine (60%) reported to be married at the time of the interviews.

Regarding their marital status, eight respondents were married, three divorced, three single, and one cohabiting. As to their occupation at the time of diagnosis/treatment, most interviewees (N = 06) were working, two on sick-leave for other health problems, four retired, two studying, and one unemployed (Table 1).

**Table 1:** Characterization of interviewees by sex, age, education, marital status, clinical form of TB, and occupation

**Int.**	**Sex**	**Age**	**Schooling**	**Marital status**	**Clinical form**	**Occupation at the time of the interviews**
1	M	73	8 to 12 years	Married	peritoneal	military, retired
2	M	40	8 to 12 years	Married	pleural	heavy-duty hydraulic technician, rodeo clown, on sick leave
3	M	58	Illiterate	Divorced	pulmonary	bricklayer, on sick leave
4	M	31	8 to 12 years	Single	pulmonary	general services assistant, working
5	M	46	4 to 7 years	Divorced	pulmonary	bricklayer, unemployed
6	M	73	1 to 3 years	Divorced	pulmonary	rectifier, retired
7	M	48	4 to 7 years	Married	pulmonary	general services assistant, working
8	M	80	12 or more years	Married	pulmonary	professor, working
9	F	19	8 to 12 years	Single	pulmonary	studying
10	F	56	4 to 7 years	Married	pulmonary	visually impaired, retired
11	M	56	8 to 12 years	Married	pleural	auto-mechanic, retired
12	F	29	8 to 12 years	Married	pulmonary	steel worker, working
13	M	16	8 to 12 years	Single	pulmonary	studying
14	M	52	8 to 12 years	Cohabiting	pulmonary	driver, working
15	M	52	12 or more years	Married	pulmonary	teacher, working

### Suffering caused by disease

In this study, suffering was primarily caused by fear of dying, of transmitting the disease to others, and of being discriminated against, which is in accordance with the literature [13]. Suffering was mainly due to stigma and ignorance about TB. However, acceptance and resilience were essential to ensure completion of treatment.

Fear of transmission, reported by the participants, is characterized by anticipatory angst of transmitting the disease to others, especially to family members (parents, children, and grandchildren). Fear of transmitting the disease led the patients to shy away from social interaction.

*It was perhaps the fear of transmitting it to others; I didn’t want them to go through what I was going through* […]*. I got really worried about my father because he’s got diabetes.* (I9)

*I gave up my bedroom; I began to sleep in the living room not to contaminate others. I had my own cup, my own plate.* (I7)

Most interviewees’ accounts indicate deficient knowledge about TB transmission modes, which contributed to their fear of transmitting the disease to others. In addition, families did not seek the necessary knowledge about the disease so as to avoid reproducing discriminatory actions and to establish procedures based on real transmission risks. This was evidenced by TB patients’ family members averting sharing objects or immediate surroundings with them.

[…] *I moved to a room in the back of the house;* [family members] *would pass me food through the window to avoid coming in my room.* (I5)

In addition to fear of infecting others, participants in this study remained isolated from friends and family for fear of being discriminated against. Thus, they hid their disease and suffered in advance by shying away from social life. In agreement with other studies [5,14,15], the suffering experienced by these respondents was primarily linked to the idea of contagion and likelihood of being discriminated against, which made them feel lonely and stigmatized.

*Nobody knew, just my family, who live with me.* […] *For this reason I was able to complete treatment without anyone knowing; only my family knew about it.* (I9)

TB was perceived by patients as a source of suffering, since they experienced social isolation, treatment-related difficulties, and changes in body image. This may be represented as loss, suffering, sadness, and anger [5]. In a Brazilian study conducted about general aspects of TB patients’ sexuality in São Paulo State, negative feelings such as fear, shame, prejudice, loneliness, and contempt were mentioned by most respondents [14].

Social isolation due to fear of transmitting the disease to others drives patients away from their families, thereby promoting further isolation and loneliness [14]. The findings of this study confirm that TB patients’ beliefs can lead to self-discrimination.

In order to avoid embarrassing situations, patients resorted to hiding their illness from work colleagues and friends, i.e., they resorted to silence when dealing with these situations. Their choosing to hide the disease from others appears justified as several studies report embarrassing situations experienced by TB patients [5,13,15].

Fear of death was also reported in other studies. According to some authors [5], physical debility caused by TB – associated with the long-standing image of incurable disease – leads patients to believe that TB is unescapably fatal and to see their death as imminent. Conversely, in the context of this study, respondents regarded treatment as a way to stay alive and healthy, which may have contributed to their adherence to treatment.

*Because when we’d hear about tuberculosis, it was said to be a deadly disease.* (I2)

*I felt that I had to do my very best not to skip a single day of treatment.* (I6)

It is thus believed that healthcare professionals should propose strategies to mitigate this suffering. Amongst possible activities is providing opportunities for groups of TB patients to discuss their condition, spaces where they can voice their fears and concerns, thereby helping them to think through their own prejudices and to find new ways of coping with their disease.

In spite of their suffering, participants in this study confronted and overpowered the disease, overcoming all obstacles to successful treatment completion. Acceptance and resilience seem to have been critical in this process.

*It’s hard, but you got to get yourself together and move on. You just can’t give up; if you succumb to the disease, things can get really complicated.* (I2)

Religious faith was reported to be essential to treatment completion. Respondents said that despite all suffering, they were able to iron out difficulties because they accepted their disease and believed that God would help them through.

*You feel so small and tall at the same time. Faith helps you through treatment.* (I1)

*But you have to take responsibility for your treatment, as I did. It wasn’t easy, but I fought hard and accepted the disease and thank God I’m cured!* (I10)

In order to be able to deal with suffering and minimize pain, other patients took charge of their own health-illness process. During treatment, they tried not to think about the disease and to move on with their lives.

[…] *I tried not to touch the subject, not to remember constantly that I had the disease*. […] *I tried to lead my life as if I didn’t have it… life as usual… to live as if everything was normal, as if I didn’t have to take any medicine; the same as before. That’s how it was for me.* (I4)

Religion appears as a means of coping with adversities such as TB [16]. This author suggests that religion – religious beliefs and practices – can be a determinant in the health-illness process, since those individuals who practice it benefit from it, by adopting healthier habits and behaviors, as evidenced in this study.

### Impact on familial and social settings

This category represents the support given by families, friends, and work colleagues, which was shown to be ambivalent. This thematic category also depicts effects of TB on patients’ work and financial situation.

Family support appears to be of paramount importance; patients’ statements suggest that it is present from the moment of diagnosis of the disease to its cure. Participants in this study reported that they had family members on whom they could depend emotionally; they also reported that this emotional support was vital to completion of TB treatment [17], since it enabled them to share and cope with difficulties posed by the disease [18].

*I think that family support counts the most in a disease like TB. No doctor, no medicine, nothing suffices. Family support is essential.* […] *So, that cheers you up; you are not forsaken. If you feel neglected, that’s the end. Thank God that didn’t happen to me!* (I1)

As to friends and colleagues that learned about the disease, the interviewees reported that not all of them were supportive. Although there are accounts of friends’ support, patients’ most common experience regarding colleagues is that of isolation, which persists even after patients return to work after medical leave.

The fact that colleagues distanced themselves from the interviewees was probably due to the formers’ ignorance about transmission modes and enduring stigma and discrimination against TB patients in society at large. In the case of participants in this study, knowing that other people they knew had had TB and had been cured promoted their adherence to treatment.

*People distance themselves from you, after they learn that it is a catchable disease; friends distance themselves a little.* (I7)

*One friend told me that he had had it and was cured. That was great; that cheered me up a little.* […] *But just friends, I only talked about it with friends. But that wasn’t always a good thing to do… you may lose a friend.* (I6)

*Tuberculosis is difficult, because people tend to shun away from you, to keep their distance. Depending on where you arrive… especially people who worked with me – they just walked away;* […] *people are afraid.* […] *When I first returned to work, people looked at me and sort of took a step back; now it’s back to normal.* (I12)

The way that family and friends relate to TB patients interferes with their everyday life. TB patients do not feel stigmatized when their spouses, families, and friends do not start acting differently [17]. This was corroborated by this study.

Families can promote patients’ adoption of healthier habits, behaviors, and attitudes leading to a successful therapeutic treatment [18]. However, [19] indicate that there is poor interaction between healthcare professionals and families, which concurs to deficient information about patient, disease, and treatment. Thus, this study suggests that healthcare teams should work together with TB patients’ families, friends, and colleagues so as to mitigate stigma and ignorance about the disease.

With regard to repercussions at the workplace, the interviewees who had jobs at the time took a medical leave from their work until treatment completion. In these cases, their salaries were paid by Brazil’s Social Security System.

*I took a leave from work;* […] *I didn’t work; I only returned to work when the treatment was over. I did nothing for six months*. (I14)

Participants attribute their need to be away from work to the distance between their workplace and the healthcare clinic, where the TB medicine was taken, as well as to their physical debility and inability to keep working.

Studies [19,20] indicate that patients deem supervised treatment as an impediment to work since it implies their daily/weekly visit to the healthcare clinic to take the medicine. This makes it difficult for them to reconcile treatment to daily activities. It should be remarked that since TB is a chronic and debilitating condition requiring lengthy treatment, it ends up causing ruptures/changes in individuals’ everyday social production and reproduction [20]. It may also be gathered from the interviews that this time away from work may have been necessary to prevent embarrassment and discrimination at the workplace.

*I stayed home for six months because* [place of work] *is in the countryside; I couldn’t come to the clinic to take the medicine. Also, it was too far for them to bring it to where I worked. So I took it at home or at the clinic. I was six months away from work.* (I7)

*Because of the treatment, you can’t work anymore. It’s really stringent, to avoid contagion.* […] *To prevent contact with people and contagion. Also, people sort of avoid you… ‘He’s sick, so let’s not go with him; let’s go with someone else…’* (I14)

Self-employed and underemployed patients are the ones that suffer the most as their income is affected. Brazilian and international studies [18,21] indicate that dismissal from work does occur after patients return from sick leave; it seems to derive from discrimination. However, regularly employed patients in this study did not lose their jobs; neither did their income drop as they kept on receiving their salaries from Social Security.

Since TB treatment in Brazil is free of charge, the impact of the disease on participants’ financial situation was irrelevant [1]. Interviewees only reported having to spend more on food as they and their families were more concerned about buying quality products.

*Ah, you end up spending more money. We spent more money because, since I was very weak, we had to buy more fruit, more wholesome food*. (I10)

### Support from health service vs. Deficient cultural knowledge

In order to complete their TB treatment, patients considered the relationship with healthcare workers vital in that it provided them with opportunities to share and cope with disease-derived difficulties. Besides emotional support, patients were also supplied with information and orientation about their health conditions.

*It was great; I was very well taken care of. The girls there* […] *helped me a lot. As you arrive there, they cheer you up, talk to you, joke around* […]; *that brightens things up a little*. (I12)

*We got there and they* [referring to healthcare professionals] *treated us like their friends* […]. *So, as to the healthcare service, the team who took care of me helped me a lot; I had no problems. I was well taken care of*. (I2)

However, despite the respondents in this study feeling well taken care of by the healthcare service and reporting to have received information, their description of the relationship with the healthcare service and staff was vague. It may be inferred that these patients did not feel comfortable enough to voice their concerns and problems during treatment, which agrees with another study [22].

The literature reports that effective communication empowers patients and families with respect to the disease and assists in changing equivocal meanings that have been constructed and internalized by different individuals throughout their life [5,22]. By crafting an emotional bond between practitioner and patient, their relationship no longer constitutes mere provision of service; it becomes more intimate; it promotes more personal conversations, many moments to share experiences. These moments allow patients more freedom to express their anxieties and doubts, which in turn helps to demystify this disease. They also alleviate patients’ anxiety and pain, which are known to affect psychological, economic, spiritual, and physical human dimensions [5,18,23,24]. It may be inferred that an emotional bond was not established with every respondent, despite its being one of the expected results of employing DOT.

Nonetheless, in spite of feeling welcomed by the healthcare service, participants in this study provided inaccurate information about the objectives of supervised treatment, modes of contagion, and basic knowledge about TB. Hence, it is evident that friends, coworkers, and family are not the only ones ill-informed about the disease. This lack of knowledge affects everyone and can be considered a feature of Brazilian culture.

[…] *I got it from someone’s cup somewhere; I don’t know. I can’t explain. I really have no idea how it starts; this stuff must be deep-rooted; something that accumulates and expands*. (I14)

[…] *because they said it’s not catchable; it’s inherited*. (I6)

Respondents stated that their illness was related to work conditions, health conditions, lifestyle habits (smoking and drinking), air quality, and even to contact with contaminated objects and utensils. It appears that in the context under investigation adjuvant clinical-epidemiological factors are considered to be of central importance as if they were etiological factors.

*Ah, I think I got it at work; there’s a cold chamber there. I used to come in and out of it all the time. I think that’s how I got it.* (I4)

*My work conditions and schedule helped to bring it about. I didn’t eat well, sleep well; then, immunity dropped, stress increased… it’s not easy*. (I12)

*The doctor said that the virus may have been there already;* [the disease] *manifested because I had lost too much weight. That’s how it may have happened.* (I1)

Lifestyle habits such as smoking and drinking were cited as probable causes of TB. However unconvinced, one of the participants believed that he had caught it because of his unhealthy lifestyle. This shows that the population under study is uninformed and/or misinformed about the difference between causal agency and co-morbidity.

*I’m not sure. I smoke and used to drink a lot. I don’t know whether it was caused by my drinking or my smoking habit, but I think it had nothing to do with that… I don’t know.* (I3)

Given that participants in this study had been in close contact with healthcare professionals for at least six months, it is worrying that they still reproduced concepts laden with prejudice and lacking in knowledge about transmission modes, especially because this fact does not seem to be related to patients’ years of schooling as ten of them had eight or more years of formal education.

On the other hand, the participants with 12 or more years of schooling stated that although TB had been incurable in the past, it was curable nowadays. They also reported concern about large concentrations of people because of increasing risk of contagion and remarked that after 15 days TB was no longer transmissible, among other pieces of correct information about the disease [1].

[…] *I think about São Paulo at rush hour… on a bus on a rainy day; everybody coughing, windows closed… I don’t know…* […] *Some say that it can lie dormant in the lungs for a long time.* (I15)

It should be remarked that in this study informative support was essential to facilitate adherence to treatment and minimize the suffering caused by distressing situations and lack of knowledge [18]. Some authors believe that greater disclosure is needed about TB, its transmission modes, signs, and symptoms so as to promote early diagnosis and prevention [18], and that these actions should not be limited to healthcare professionals [25].

It is important to devise educational programs directed to society at large as well, addressing different aspects related to TB treatment and patients. They should promote patients’ and their caretakers’ health and safety [26], thereby increasing the community’s scientific literacy. However, increasing patients’ scientific literacy should not be limited to physicians’ teachings, concepts of TB etiology and contagion, and representations of the health-illness process. There is a wide array of possibilities and explanations [4].

In a study conducted in the same region of São Paulo State, nurses denounce deficient training of healthcare professionals as one of the weaknesses of the local *Programa de Controle da Tuberculose* (Tuberculosis Control Program). It should be remarked that this deficiency may delay diagnosis and, as a result, increase the risk of transmission, thereby compromising the success of this program [27].

However, the success of PNCT is determined by the following conditions: human relationships, empathy, and the bond established among health professionals and the target population, as well as the patients’ willingness to express their anguish and report their needs [1]. However, some Brazilian studies show that health professionals still find it difficult to incorporate interventions that take into account TB patients’ unique realities into their work process [28].

### Implications to Patients’ post-treatment life

The experience of having been through TB may have several ramifications. The way the health-illness process is understood by individuals is paramount to their perception and to the emergence of these implications.

The analysis of the data suggests that after treatment completion most patients’ personal and professional life returned to what it had been before the disease. Notwithstanding, some participants cite changes in different spheres of their life. It seems that Brazilian people are immersed in a psycho-culture with a broad and heterogeneous spectrum, which gives rise to markedly polysemic meanings.

*It’s back to normal.* […] *I came out of it unscathed; I’m free*. […] *My life’s back to normal; it’s normal*. (I2)

*Nothing’s changed in my life after TB; in spite of the disease, I kept on working, making plans, living in high spirits. I kept studying, which is what I like doing*. (I8)

Negative post-treatment implications are generally related to physical issues. However, having gone through a serious illness such as TB, with all its implications, enabled the respondents to reflect on their own health. Although studies indicate that these effects are limited to changes in lifestyle and health conditions, it should be emphasized that changes at the psychosocial level were also identified in the participants’ answers. At the mention of the disease and the changes caused by it, it appears that TB has left ever-lasting impressions in their life, especially because of the suffering involved in the course of the disease.

*It’s just that I feel more tired now that the treatment is over. I feel more worn-out; I still feel weak; I’m often indisposed*. […] *I don’t have the same energy as I did before.* (I12)

*I still have an upset stomach*. (I8)

*It made no difference in the past. I could stay up all night long; in winter, in summer… I can’t do that anymore; I take more care now*. (I11)

*I quit smoking; you become more mindful of your health; you worry more about hygiene… That’s it: you take more care of your own health*. (I14)

A Brazilian study on social representations about living with TB confirms that the disease diagnosis changed their patients’ lives. These changes, however laden with negative perceptions, were not always spoken about directly; they were subliminally understood from patients’ accounts of their pre- and post-TB lives [29], which corroborate the study in question.

## Conclusions

This study only considers the perspective of patients who completed the treatment, which may lead to biased results. However, eliciting the views of individuals that have been able to overcome all the obstacles posed by TB treatment and resume their daily lives can shed light on another facet of this complex disease.

Ignorance about TB, along with stigma, suffuses all of the thematic units in this study. People suffer from and are victims of prejudice due to continuing stigma and lack of knowledge about the disease on the part of patients themselves and society at large.

Social support is also influenced by unfamiliarity and negative preconceptions about TB, since distancing of friends and colleagues was reported by the participants. It is evident that those going through this illness and those who are not are bundled together in the same large group of individuals immersed in TB myth and pseudoscience. Although patients and non-patients’ bodies may experience TB differently, their understanding of clinical phenomena does not seem to differ substantially.

It was noted that the stated criteria for being on medical leave from work during treatment consisted of a combination of epidemiological and sociological reasons, which did not always seem clear to patients.

To be sure, TB caused changes in the respondents’ lives. Notwithstanding, after treatment, they reported to have resumed their daily routines. They also reported that small physical changes had persisted and that habits had changed after treatment; they began to take their health more seriously. It also was found that the TB-related suffering still lingers after treatment and cure; it resurfaced when the participants spoke about the disease.

Acknowledging the emotional distress caused by TB throughout its course, which persists even after treatment, is essential to bring about much-needed changes in healthcare. It is up to healthcare professionals, in line with the DOTS strategy, to use empowerment strategies and create opportunities to discuss the disease with patients and families in order to break the vicious cycle of misinformation and prejudice.

## Competing interests

The authors declare that they have no competing interests.

## Authors’ contributions

AALD contributed to the study design, data collection, and qualitative analysis as well as to preparing the draft manuscript. DFO contributed to the fieldwork. ERT contributed to the study methodology and interpretation of results. RMF contributed to the study conception, design, qualitative analysis of data, and to preparing the draft manuscript. All authors contributed to the interpretation of results and drafting of the article. All authors have read and approved the final manuscript for submission.

## Pre-publication history

The pre-publication history for this paper can be accessed here:

http://www.biomedcentral.com/1471-2458/13/595/prepub

## References

[B1] Brasil. Ministério da Saúde. Secretaria de Vigilância em Saúde Manual de Recomendações para o Controle da Tuberculose no Brasil. Programa Nacional de Controle da Tuberculose. [Brazil. Ministry of Health, Secretariat of Health Surveillance. Manual of Practice for the Control of Tuberculosis in Brazil] 2010 Brasília: National Program for Tuberculosis Control

[B2] World Health Organization Global Tuberculosis Control 2010 Geneva: WHO Report 2010

[B3] Porto A Social representations of tuberculosis: stigma and prejudice Rev Saude Publica 2007 41 suppl. 1 43 49 18038090 10.1590/s0034-89102007000800007

[B4] Gonçalves H Costa JSD Menezes AMB Knauth D Leal OF Adesão à terapêutica da tuberculose em Pelotas, Rio Grande do Sul: na perspectiva do paciente. [Adherence of tuberculosis in Pelotas, Rio Grande do Sul: the patient’s perspective] Cad Saude Publica 1999 15 4 777 787 10.1590/S0102-311X1999000400012 10633200

[B5] Souza SS Silva DMGV Meirelles BHS Social representations of tuberculosis Acta Paul Enferm 2010 23 1 23 28 10.1590/S0103-21002010000100004

[B6] Goffman E Stigma: notes on the management of spoiled identity 1986 New York: Touchstone

[B7] Queiroz EM Bertolozzi MR Tuberculosis: supervised treatment in North, West and East Health Departments of São Paulo Rev esc enferm USP 2010 44 2 453 461 [http://www.scielo.br/pdf/reeusp/v44n2/en_30.pdf] 10.1590/S0080-62342010000200030 20642060

[B8] Mazzei AMA Monroe AA Sassaki CM Gonzales RIC Villa TCS Suporte social para portador de tuberculose no serviço de saúde e na comunidade. [Social support to tuberculosis patients in the health service and the community] Bol Pneumol Sanit 2003 11 2 41 46

[B9] Turato ER Tratado de metodologia da pesquisa clínico qualitativa: construção teórico-epistemológica, discussão comparada e aplicação nas áreas da saúde e humanas [Treaty of clinical qualitative research methodology: theoretical-epistemological comparative discussion and application in the areas of health and human sciences] 2011 5 Vozes: Petrópolis

[B10] Minayo MCS O desafio do conhecimento: pesquisa qualitativa em saúde [The challenge of knowledge: qualitative health research] 2010 Hucitec: São Paulo

[B11] Bassora JB Campos CJG Metodologia clínico-qualitativa na produção científica no campo da saúde e ciências humanas: uma revisão integrativa. [Clinical-qualitative methodology in scientific production in health and human sciences: an integrative review] Rev Eletr Enf 2010 12 4 753 760 [http://www.fen.ufg.br/revista/v12/n4/v12n4a22.htm]

[B12] Bardin L Análise de Conteúo [Content Analysis] 2010 Lisboa: Edições 70

[B13] Baral SC Karki DK Newell JN Causes of stigma and discrimination associated with tuberculosis in Nepal: a qualitative study BMC Publ Health 2007 7 211 [http://www.biomedcentral.com/1471-2458/7/211] 10.1186/1471-2458-7-211 PMC2018718 17705841

[B14] Bertazone EC Gir E Aspectos gerais da sexualidade dos portadores de tuberculose pulmonar atendidos em unidades básicas de saúde de Ribeirão Preto-SP. [The sexuality of people with pulmonary tuberculosis cared at health basic units at Ribeirão Preto-SP] Rev latino-am enfermagem 2000 8 1 115 122 10.1590/s0104-11692000000100016 10909386

[B15] Rundi C Understanding tuberculosis: perspectives and experiences of the people of Sabah, East Malaysia J Health Popul Nutr 2010 28 2 114 123 [http://www.ncbi.nlm.nih.gov/pmc/articles/PMC2980872] 20411673 10.3329/jhpn.v28i2.4880 PMC2980872

[B16] Bousso RS Poles K Serafim TS Miranda MG Religious beliefs, illness and death: family’s perspectives in illness experience Rev esc enferm USP 2011 45 2 397 403 [http://www.scielo.br/pdf/reeusp/v45n2/en_v45n2a13.pdf] 10.1590/S0080-62342011000200014 21655790

[B17] Rintiswati N Mahendradhata Y Suharna Susilawati Purwanta Subronto Y Varkevisser CM van der Werf MJ Journeys to tuberculosis treatment: a qualitative study of patients, families and communities in Jogjakarta, Indonesia BMC Publ Health 2009 9 158 [http://www.biomedcentral.com/1471-2458/9/158] 10.1186/1471-2458-9-158 PMC2700804 19473526

[B18] Souza SS Silva DMGV Passando pela experiência do tratamento para tuberculose. [Experiencing treatment for tuberculosis] Texto Contexto Enferm 2010 19 4 636 643 10.1590/S0104-07072010000400005

[B19] Nogueira JA Trigueiro DRSG Sá LD Silva CA Oliveira LCS Villa TCS Scatena LM Family focus and community orientation in tuberculosis control Rev Bras Epidemiolol 2011 14 2 207 216 10.1590/S1415-790X2011000200003 21655688

[B20] Campinas LLSL Almeida MMMB Agentes Comunitários de Saúde e o acolhimento aos doentes com tuberculose no Programa Saúde da Família Bol Pneumol Sanit 2004 12 3 145 154

[B21] Sagbakken M Frich JC Bjune G Barriers and enablers in the management of tuberculosis treatment in Addis Ababa, Ethiopia: a qualitative study BMC Publ Health 2008 8 11 [http://www.biomedcentral.com/1471-2458/8/11] 10.1186/1471-2458-8-11 PMC2257959 18186946

[B22] Lewis CP Newell JN Improving tuberculosis care in low income countries – a qualitative study of patients’ understanding of “patient support” in Nepal BMC Publ Health 2009 9 190 [http://www.biomedcentral.com/1471-2458/9/190] 10.1186/1471-2458-9-190 PMC2703637 19534811

[B23] Paz EPA Sá AMM The daily routine of patients in tuberculosis treatment in basic health care units: a phenomenological approach Rev Latino-Am Enfermagem 2009 17 2 180 186 [http://www.scielo.br/pdf/rlae/v17n2/pt_07.pdf] 10.1590/S0104-11692009000200007 19551270

[B24] Gomes ALC Sa LD The concepts of bonding and the relation with tuberculosis control Rev esc enferm USP 2009 43 2 365 372 [http://www.scielo.br/pdf/reeusp/v43n2/en_a16v43n2.pdf] 10.1590/S0080-62342009000200016 19655678

[B25] Muñoz Sánchez AI Bertolozzi MR Percepción de los trabajadores de salud de unidades básicas de salud de São Paulo (Brasil) sobre la tuberculosis Av enferm 2009 27 2 19 24 [http://www.enfermeria.unal.edu.co/revista/articulos/xxvii2_3.pdf]

[B26] Bertazone EC Gir E Hayashida M Situações vivenciadas pelos trabalhadores de enfermagem na assistência ao portador de tuberculose pulmonar. [Nursing workers’ experiences in care for pulmonary tuberculosis patients] Rev latino-am enfermagem 2005 13 3 374 381 10.1590/s0104-11692005000300012 16059543

[B27] Caliari JS Figueiredo RM Tuberculosis: patient profile, service flowchart, and nurses’ opinions Acta paul enferm 2012 25 1 43 47 [http://www.scielo.br/pdf/ape/v25n1/en_v25n1a08.pdf] 10.1590/S0103-21002012000100008

[B28] Trigueiro JVS Silva ACO Gois GAS Almeida SA Nogueira JA Sá LD Percepção de enfermeiros sobre educação em saúde no controle da tuberculose. [Nurse’s view on health education for the tuberculosis control] Cienc Cuid Saude 2009 8 4 660 666

[B29] Souza SS Representações sociais sobre o viver com tuberculose [Social representations about living with tuberculosis]. Dissertation 2006 Federal University of Santa Catarina, Florianópolis, Brazil: Graduate Program in Nursing

